# *Bartonella* spp. and *Coxiella burnetii* Associated with Community-Acquired, Culture-Negative Endocarditis, Brazil

**DOI:** 10.3201/eid2108.140343

**Published:** 2015-08

**Authors:** Rinaldo Focaccia Siciliano, Jussara Bianchi Castelli, Alfredo Jose Mansur, Fabiana Pereira dos Santos, Silvia Colombo, Elvira Mendes do Nascimento, Christopher D. Paddock, Roosecelis Araújo Brasil, Paulo Eduardo Neves Ferreira Velho, Marina Rovani Drummond, Max Grinberg, Tania Mara Varejao Strabelli

**Affiliations:** University of São Paulo Medical School, São Paulo, Brazil (R.F. Siciliano, J.B. Castelli, A.J. Mansur, M. Grinberg, T.M.V. Strabelli);; Adolfo Lutz Institute, São Paulo (F. Pereira dos Santos, S. Colombo, E.M. Nascimento, R.A. Brasil);; Centers for Disease Control and Prevention, Atlanta, Georgia, USA (C.D. Paddock);; State University of Campinas Medical School, Campinas, Brazil (P.E.N.F. Velho, M.R. Drummond)

**Keywords:** endocarditis, *Bartonella*, *Coxiella burnettii*, Brazil, zoonoses, bacteria

## Abstract

We evaluated culture-negative, community-acquired endocarditis by using indirect immunofluorescent assays and molecular analyses for *Bartonella* spp. and *Coxiella burnetii* and found a prevalence of 19.6% and 7.8%, respectively. Our findings reinforce the need to study these organisms in patients with culture-negative, community-acquired endocarditis, especially *B. henselae* in cat owners.

Worldwide, *Bartonella* spp. and *Coxiella burnetii* endocarditis have varied prevalences and clinical effects (*1*,*2*). Detection is difficult in routine blood cultures, so different diagnosis methods are needed. Our study investigated the frequency of and the risk factors for *Bartonella* spp. and *C. burnetii* infection in cases of culture-negative, community-acquired endocarditis.

## The Study

During January 2004–January 2009, the Infection Control Team from the university hospital at São Paulo, Brazil (Instituto do Coração–Hospital das Clínicas da Faculdade de Medicina da Universidade de São Paulo) used active surveillance to identify 369 patients with endocarditis. The study focused on community-acquired endocarditis caused by fastidious bacteria. Patients >18 years of age with confirmed endocarditis were included as a prospective inception cohort of patients (*3*). Excluded were patients with health care–associated endocarditis (i.e., patients with prosthetic valve endocarditis in the first postoperative year, hemodialysis patients, and nosocomial endocarditis patients) (*4*).

Indirect immunofluorescence assays (IFAs) were performed for all patients with negative blood cultures <7 days after admission at a referral center for rickettsial infections (Adolfo Lutz Institute, São Paulo). The same observer analyzed all assays; IgG titers ≥1:800 for *B. henselae* and *B. quintana* (*5*) and anti–phase I IgG titers ≥1:800 for *C. burnetii* (*6*) were considered positive (Technical Appendix). New diagnostic criteria for Q fever endocarditis were used (*6*).

Immunohistochemical and molecular methods were applied to valve tissue specimens and serum samples of patients whose serum samples were positive for *Bartonella* spp. or *C. burnetii*. DNA from paraffin-embedded valve tissue specimens and serum samples were extracted. Samples positive for *Bartonella* by IFA were analyzed by using 5 different PCRs to 4 distinct regions. Tissue and serum DNA from patients positive for *C. burnettii* by IFA were tested by quantitative PCR (Technical Appendix Table 1) (*7*).

Of the 369 identified endocarditis patients, 221 (59.9%) were included in the study; median age of included patients was 53 years. Of included patients, 144 (65.2%) were male; 107 (48.4%) had prosthetic valves; 209 (94.6%) had left-sided endocarditis; and 152 (68.8%) had concurrent conditions. Of patients with concurrent conditions, 62 (40.8%) had hypertension, 17 (11.2%) had diabetes, 53 (34.9%) had heart failure, and 36 (23.7%) had other conditions. Of the 221 patients included in the study, microorganisms were identified in 170 (76.9%); specimens from 51 (23.1%) patients were culture negative. 

A standardized questionnaire regarding exposure to cats, ectoparasites, or farm animals was administered to patients with culture-negative endocarditis. For the 170 samples in which microorganisms were found, the most commonly identified bacteria were *viridans*-type *Streptococci* (81 [47.6%]), *Streptococcus bovis* (17 [10.0%]), *S. pneumonie* (6 [3.5%]), *S. agalactiae* (2 [1.2%]), *S. pyogenes* (2 [1.2%]), *Enterococcus fecalis* (13 [7.6%]), *E. faecium* (3 [1.8%]), other enterococci (4 [2.4%]) and *Staphylococcus aureus* (14 [8.2%]).

For the 221 patients in the study, findings from 10 (4.5%; 95% CI 3.96%–5.09%) patients (Figure) showed *Bartonella* spp., and 4 (1.8%; 95% CI 1.58%–2.04%) showed *C. burnetii* endocarditis. For the 51 culture-negative endocarditis patients, *Bartonella* spp. was found in cultures from 10 (19.6%; 95% CI 9.8%–33.1%), and *C. burnetii* was found in 4 (7.8%; 95% CI 2.2%–18.9%). The Table shows the immunohistochemical and molecular biology analyses for patients with positive IFA results. *Bartonella* spp. DNA was detected with >1 PCR in all 6 patients whose paraffin-embedded valve tissue samples were found positive for *Bartonella* spp. For the other 4 patients with *Bartonella* spp., DNA was detected in 2 serum samples. Amplicons were sequenced, and their analyses showed that the cultures from 2 patients had 100% similarity with *B. quintana* (GenBank accession no. BX897700.1); cultures from 4 patients had 100% similarity with *B. henselae* infection (GenBank accession no. BX897699.1). Cultures from 2 patients were positive for *Bartonella* spp. by using IFA but negative by using PCR. 

**Figure:**
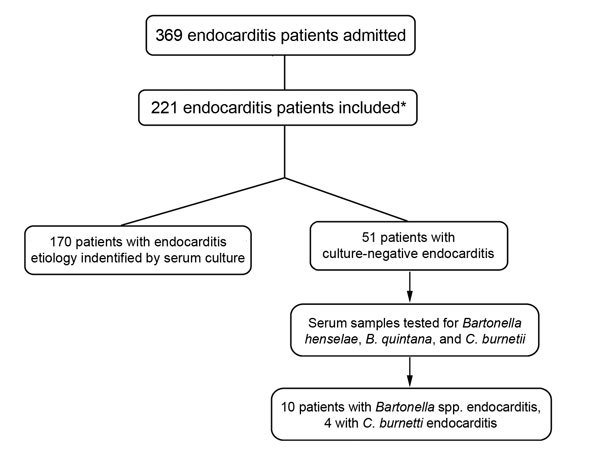
Distribution of patients etiologically diagnosed with endocarditis and admitted to the heart institute (Instituto do Coração) at the University of São Paulo Medical School, Sao Paulo, Brazil, January 2004–January 2009. *A modified Duke criteria (*3*) was used to determine inclusion of 221 patients. Excluded were 148 patients: 58 with unconfirmed endocarditis, 28 with endocarditis caused by cardiac implantable electronic devices, 47 with nosocomial endocarditis, and 15 hemodialysis patients.

**Table:** Serologic, immunohistopathologic, and molecular test results for patients with infective endocarditis caused by *Bartonella* spp. or *Coxiella burnetii*, Brazil*

Patient no., by infection type	Serum IgG ≥800 by IFA†	Immunohistochemical analysis of cardiac valve vegetation		Microorganism by histologic analysis	PCR‡	Species of *Bartonella*
		*Bartonella spp.*	*C. burnetii*			
*Bartonella* spp.						
1	+	+	Neg	Gram-negative coccobacilli	+	*B. quintana*
2	+	+	Neg	Gram-negative coccobacilli	+	*B. henselae*
3	+	+	Neg	None	+	*B. henselae*
4	+	NA	NA	NA	Neg	NA
5	+	+	Neg	Gram-negative coccobacilli	+	NA
6	+	+	Neg	Gram-negative coccobacilli	+	*B. quintana*
7	+	NA	NA	NA	+	*B. henselae*
8	+	NA	NA	NA	+	NA
9	+	NA	NA	NA	Neg	NA
10	+	+	Neg	Gram-negative cocci	+	*B. henselae*

*C. burnetii*	Serum anti–phase I IgG ≥800 by IFA§	Immunohistochemical analysis of cardiac valve vegetation		Microorganism by histologic analysis	PCR‡	Q fever endocarditis (*6*)
		*Bartonella spp.*	*C. burnetii*			
11	+	Neg	+	Small gram-negative coccobacilli	+	Definite 2A criteria
12	+	Neg	+	None	+	Definite 2A criteria
13	+	Neg	+	None	+	Definite 2A criteria
14	+	NA	NA	NA	+	Definite 2B criteria¶

*IFA, immunofluorescence assay; +, positive; Neg, negative; NA, material not available for analysis. ¶Serologic result >6,400 and vegetation on ecocardiography. †*B. henselae *or *B. quintana.* ‡Serum or tissue sample. §*C. burnetii.*

All patients used antimicrobial drugs for 7 days before sample collection. All endocarditis patients whose cultures were found to be positive for *C. burnetii* by using IFA were also positive by using quantitative PCR: 3 by serum samples and 2 by paraffin-embedded valve tissue specimens (Technical Appendix Table 2).

Clinical and follow-up findings from *Bartonella* spp. and *C. burnetii* endocarditis patients are shown in Technical Appendix Table 3. *Bartonella* spp. infection was associated with low levels of C-reactive protein on admission and chronic symptoms related to endocarditis (Technical Appendix Table 4). Three (75%) of 4 patients with *Bartonella henselae* endocarditis were associated with a cat living in the patient’s home, compared with 6 (12.8%) of 47 patients with culture-negative *Bartonella henselae* negative endocarditis (p = 0.015 by Student *t*-test). Hydroxychloroquine was unavailable in our facility; therefore, we used a second-line therapy for *C. burnetii* endocarditis. Hydroxychloroquine was replaced with ciprofloxacin, and treatment was extended for 72 months (*8*). Subsequently, symptoms resolved, and antibody titers reduced substantially, considered a favorable response (*9*) (Technical Appendix Table 3).

## Conclusions

In this study, the systematic use of IFA detected a 4.5% (10/221) prevalence of community-acquired endocarditis due to *Bartonella* spp. and a 1.8% (4/221) prevalence due to *C. burnetii*. For the 51 culture-negative endocarditis patients, IFA enabled recognition of the endocarditis etiology in 14 (27.5%) patients (*Bartonella* spp. in 10 [19.6%] and *C. burnetii* in 4 [7.8%]). Some of these patients have been recognized as having the first cases of endocarditis caused by these microorganisms in Brazil (*10*,*11*).

Prevalences of *Bartonella* spp. endocarditis vary worldwide by region studied (*1*). In a broad series of 759 culture-negative endocarditis patients in France, serum samples showed high sensitivity for detection of *C. burnetii* and *Bartonella* spp. infections, compared with other diagnostic tools, such as PCR, cell culture, and immunohistochemical analysis (*2*). In Brazil, studies of *Bartonella* spp. infection among culture-negative endocarditis patients have shown varied results. A retrospective case series of 51 surgically treated, culture-negative endocarditis patients found 2 cases of *Bartonella* spp. and 1 case of *C. burnetii* by using PCR on valvular tissue (*12*). Another series of 46 culture-negative endocarditis patients from the city of São Paulo used PCR to investigate *Bartonella* spp. in blood and found 13 (28%) patients with positive results (*13*).

We found an association between *B. henselae* endocarditis and the presence of a cat living at a patient´s home, a risk factor indicating that clinicians should consider this infection when assessing endocarditis patients. The relatively small sample of patients with endocarditis caused by *Bartonella* spp. and *C. burnetii* limited the statistical analyses of factors associated with these infections. Serologic investigations of infections by these agents were applied only to patients with negative cultures. Although rare (*2*,*14*), co-infection by these microorganisms in culture-positive endocarditis is possible, so frequency of *Bartonella* spp. and *C. burnetii* infections in these patients may be higher than shown. Our study indicates that systematic serologic research for *Bartonella* spp. and *C. burnetii* in community-acquired, culture-negative endocarditis may be clinically useful, particularly in screening for *B. henselae* in cat owners.

**Technical Appendix.** Investigative methods and findings for patients diagnosed with endocarditis and admitted to the heart institute (Instituto do Coração) at the University of Sao Paulo Medical School, Sao Paulo, Brazil, January 2004–January 2009.
