# Supplementary material for: Bartonella spp. and Coxiella burnetii Associated with Community-Acquired, Culture-Negative Endocarditis, Brazil
Source: Emerg Infect Dis. 2015 Aug;21(8):1429–32. doi: 10.3201/eid2108.140343 (PMC4517744; doi:10.3201/eid2108.140343)
Supplement: Supplementary file 1 — Technical Appendix. Investigative methods and findings for patients diagnosed with endocarditis and admitted to the heart institute (Instituto do Coração) at the University of Sao Paulo Medical School, Sao Paulo, Brazil, January 2004–January 2009. [file 14-0343-Techapp-s1.pdf]

Article DOI: http://dx.doi.org/10.3201/eid2108140343

Bartonella spp. and *Coxiella burnetii* Associated with Community-Acquired, Culture-Negative Endocarditis, Brazil

Technical Appendix

Indirect Immunofluorescence Assays

As recommended (*1*), we used the following antigens: *Bartonella henselae* RA2552, Lot 08–0039; *Bartonella quintana,* RA2551, Lot 08–0038 obtained from the Centers for Disease Control and Prevention (Atlanta, GA, USA). We also used *C. burnetii* antiphase I (SCIMEDX Corporation, Denville, NJ, USA).

Immunohistochemical Analyses

The immunohistochemical analysis for *Bartonella henselae* was performed by Biocare Medical (Concord, CA, USA; clone H2A10). That for *Bartonella quintana* and *Coxiella burnetii* was performed by the Rickettsial Zoonoses Branch, Centers for Disease Control and Prevention.

Molecular Methods

Molecular tests were used to confirm the serologic results and identify *Bartonella* species. From 10 *Bartonella* spp. positive patients, the serum was subjected to DNA extraction by using QIAamp DNA Mini Kit (QIAGEN, Valencia, CA, USA). From 4 patients whose cultures were positive for *coxiella*, nucleic acid was extracted by serum samples by using PureLink Viral RNA/DNA Mini Kit-Life Technologies (Invitrogen, Grand Island, NY, USA). We also performed DNA extraction from formalin-fixed, paraffin–embedded valve tissue specimens from 6 patients with specimens positive for *Bartonella* spp. and 3 positive for *C. burnetii* by using PureLink Genomic DNA Mini Kit - Life Technologies (Invitrogen).

Screening of samples was performed by using 3 *Bartonella* genus-specific single tube PCR and 1 nested PCR specific for *B. henselae*; amplicons generated were sequenced. Formalin–fixed, paraffin–embedded valve tissue specimens were also submitted to real-time PCR to detect *Bartonella* spp. (*glt*A). Serum samples and formalin-fixed, paraffin–embedded valve tissue specimens of patients whose results were positive for *C. burnetii* by immunofluorescence assay

were submitted to real-time PCR to detect *C. burnetii* (IS*1111*). The molecular methods used follow the protocol described in the Technical Appendix Table 1.

References

1. Fournier PE, Mainardi JL, Raoult D. Value of microimmunofluorescence for diagnosis and follow-up of *Bartonella* endocarditis. Clin Diagn Lab Immunol. 2002;9:795–801. PubMed

2. Diniz PP, Maggi RG, Schwartz DS, Cadenas MB, Bradley JM, Hegarty B, et al. Canine bartonellosis: serologic and molecular prevalence in Brazil and evidence of co-infection with *Bartonella henselae* and *Bartonella vinsonii* subsp. *berkhoffii*. Vet Res. 2007;38:697–710. PubMed http://dx.doi.org/10.1051/vetres:2007023

3. Pitassi LH, de Paiva Diniz PP, Scorpio DG, Drummond MR, Lania BG, Barjas-Castro ML, et al. *Bartonella* spp. bacteremia in blood donors from Campinas, Brazil. PLoS Negl Trop Dis. 2015;9:e0003467 http://dx.doi.org/10.1371/journal.pntd.0003467. PubMed

4. Diaz MH, Bai Y, Malania L, Winchell JM, Kosoy MY. Development of a novel genus-specific real-time PCR assay for detection and differentiation of *Bartonella* species and genotypes. J Clin Microbiol. 2012;50:1645–9. PubMed http://dx.doi.org/10.1128/JCM.06621-11

5. Kawasato KH, de Oliveira LC, Velho PE, Yamamoto L, Del Negro GM, Okay TS. Detection of *Bartonella henselae* DNA in clinical samples including peripheral blood of immune competent and immune compromised patients by three nested amplifications. Rev Inst Med Trop Sao Paulo. 2013;55:1–6. PubMed http://dx.doi.org/10.1590/S0036-46652013000100001

6. Molia S, Chomel BB, Kasten RW, Leutenegger CM, Steele BR, Marker L, et al. Prevalence of *Bartonella* infection in wild African lions (*Panthera leo*) and cheetahs (*Acinonyx jubatus*). Vet Microbiol. 2004;100:31–41. PubMed http://dx.doi.org/10.1016/j.vetmic.2004.01.007

7. Schneeberger PM, Hermans MH, van Hannen EJ, Schellekens JJ, Leenders AC, Wever PC. Real-time PCR with serum samples is indispensable for early diagnosis of acute Q fever. Clin Vaccine Immunol. 2010;17:286–90. PubMed http://dx.doi.org/10.1128/CVI.00454-09

Technical Appendix Table 1. PCR types and references used in *Bartonella* spp. and *C. burnetii* analysis, Brazil*

Application	Target gene	Reference
Bartonella spp. conventional PCR (ITS 1)	16S–23S rRNA gene ITS	(*2*)
Bartonella spp. conventional PCR (ITS 2)	16S–23S rRNA gene ITS	(*3*)
Bartonella spp. conventional PCR (srA)	Transfer–mRNA (ssrA)	(*4*)
Bartonella henselae nested PCR (FtsZ)	Cell division protein *FtsZ*	(*5*)
Bartonella spp. real-time PCR (gltA)	Citrate synthase gene	(*6*)
C. burnetii real-time PCR (IS*1111*)	Insertion element	(*7*)

*ITS, intergenic transcribed spacer.

Technical Appendix Table 2. Serologic titles and PCR results for patients with infective endocarditis caused by *Bartonella* spp. or *C. burnetii**

Bartonella Cases	Serology (IFA) IgG			PCR (tissue or serum)					
				Bartonella spp.					*C. burnetii*
	B. henselae	*B. quintana*	Antiphase I *C. burnetii*	ITS 1	ITS 2	ssra	FtsZ	gltA	IS*1111*
1	≥1,600	≥1,600	<800	+	+	+	Neg	Neg	NP
2	≥1,600	≥1,600	<800	Neg	+	+	+	+	NP
3	≥1,600	≥1,600	<800	Neg	+	+	+	+	NP
4	≥1,600	≥1,600	<800	Neg	Neg	Neg	Neg	NP	NP
5	≥1,600	≥1,600	<800	Neg	+	Neg	Neg	+	NP
6	≥1,600	≥1,600	<800	Neg	+	+	Neg	Neg	NP
7	≥1,600	≥1,600	<800	Neg	+	Neg	+	NP	NP
8	800	<800	<800	Neg	+	Neg	Neg	NP	NP
9	≥1,600	≥1,600	<800	Neg	Neg	Neg	Neg	NP	NP
10	800	≥1,600	<800	Neg	+	Neg	Neg	Neg	NP
11	<800	<800	25,600	NP	NP	NP	NP	NP	+
12	<800	<800	6,400	NP	NP	NP	NP	NP	+
13	<800	<800	25,600	NP	NP	NP	NP	NP	+
14	<800	<800	51,200	NP	NP	NP	NP	NP	+

*IFA, immunofluorescence assay; ITS, intergenic transcribed spacer; +, positive; Neg, negative; NP, not performed.

Publisher: CDC; Journal: Emerging Infectious Diseases
Article Type: Dispatch; Volume: 21; Issue: 8; Year: 2015; Article ID: 14-0343
DOI: 10.3201/eid2108.140343; TOC Head: Dispatch

Technical Appendix Table 3. Clinical and evolving characteristics of 14 patients with endocarditis caused by *Bartonella* spp. and *C. burnettii*, Brazil*

Patients by infection type	Age, y/sex	Epidemiology	Valve/position	Antimicrobial drug treatment (d) †	Endocarditis-related complications	Surgical treatment	Cause of death	*C. burnetii* serology: At diagnosis	*C. burnetii* serology: At end of treatment
Bartonella spp.									
1	35/M	Flea	Prosthesis/aortic	Oxacillin + ceftriaxone (30) Gentamicin (17)	–	Yes	NA	Neg	
2	65/M	Domestic cat	Native/aortic	Oxacillin + ceftriaxone (19) Gentamicin (11)	Paravalvular abscess	Yes	Heart failure	Neg	
3	52/M	Domestic cat; cat scratch	Prosthesis/aortic	Oxacilin + Penicillin (5)	–	No	Malignant tachyarrhythmia	Neg	
4	31/F	Domestic cat; cat scratch	Prosthesis/mitral	Oxacillin + ceftriaxone (42)	–	No	NA	Neg	
5	60/M	Domestic cat	Native/aortic	Ceftriaxone (45) Gentamicin (30)	Paravalvular abscess + fistula	Yes	NA	Neg	
6	58/M	Homelessness	Native/aortic	Oxacillin + ceftriaxone (42) Gentamicin (30)	–	Yes	NA	Neg	
7	70/M	Domestic cat	Native/aortic	Penicillin (24)+ Gentamicin (24)	CNS emboli + Paravalvular abscess	No	Heart failure and septic shock	Neg	
8	41/M	Lice; scabies	Prosthesis/aortic	Oxacillin + penicillin (62) Gentamicin (28)	Paravalvular abscess	Yes	NA	Neg	
9	21/M	Domestic cat	Native/mitral	Penicillin (28) Gentamicin (14)	–	No	NA	Neg	
10	51/M	Homelessness	Native/aortic	Ceftriaxone (30) Gentamicin (3)	–	Yes	Heart failure	Neg	
C. burnetii									
11	41/M	Rural residence; consumption of raw milk	Native/mitral	Ciprofloxacin + doxycycline*	Heart failure	Yes	Septic shock caused by nosocomial pneumonia (day 5 after cardiac surgery and day 26 after start of *C. burnetii* treatment)	25,600	NP
12	45/M	Rural residence; consumption of raw milk	Prosthesis/aortic	Ciprofloxacin + doxycycline*	Heart failure	Yes	NA	6,400	<800
13	34/F	Rural residence; consumption of raw milk	Prosthesis/mitral	Ciprofloxacin + doxycycline*	–	Yes	NA	25,600	3,200
14	64/M	Rural residence; consumption of raw milk	Prosthesis/aortic	Ciprofloxacin + doxycycline*	–	No	NA	51,200	12,800

*–, no identified complications; NA, not applicable (patient did not die); Neg, negative; NP, not performed (patient died before end of treatment.
†Hydroxychloroquine was unavailable; second-line treatment for *C. burnettii* endocarditis (ciprofloxacin and doxycycline for 72 mos.) was used.

Publisher: CDC; Journal: Emerging Infectious Diseases
Article Type: Dispatch; Volume: 21; Issue: 8; Year: 2015; Article ID: 14-0343
DOI: 10.3201/eid2108.140343; TOC Head: Dispatch

Technical Appendix Table 4. Distribution of clinical, laboratory, and echocardiographic features of 221 patients with community-acquired endocarditis, according to whether *Bartonella* spp. infection was involved*

Characteristic	*Bartonella* spp. endocarditis no. (%)	Non–*Bartonella* spp. endocarditis no. (%)	*p* value
Male sex	9 (10)	135 (64.0)	0.092
Age ≥60 y	3 (30)	87 (41.2)	0.480
Body mass index ≥25 kg/m^2	1 (10)	71 (37.2)	0.081
Concurrent conditions	8 (80)	144 (68.2)	0.433
Valvular heart disease	7 (70)	177 (83.9)	0.250
Previous endocarditis	1 (10)	25 (11.8)	0.859
Duration of symptoms ≥30 d	8 (80)	95 (45.2)	0.048
Fever	9 (90)	188 (89.5)	0.992
≥1affected valve	0 (0)	17 (8.1)	NA
C-reactive protein ≥80 mg/L	2 (25)	90 (62.5)	0.032
Severe sepsis	4 (40)	76 (36.0)	0.798
Moderate or severe valvular regurgitation	8 (80)	153 (73.2)	0.634
Vegetation on echocardiography	9 (90)	162 (77.5)	0.351
Glomerulonephritis	1 (10)	43 (24.9)	0.285

**p* value determined by Pearson χ^2 or Fisher exact test; NA, not applicable.
